# Damage Characterization of Bio and Green Polyethylene–Birch Composites under Creep and Cyclic Testing with Multivariable Acoustic Emissions

**DOI:** 10.3390/ma8115382

**Published:** 2015-11-02

**Authors:** Alencar Bravo, Lotfi Toubal, Demagna Koffi, Fouad Erchiqui

**Affiliations:** 1 Laboratory of mechanics and eco-materials, University of Quebec at Trois-Rivières, 3351, boul. des Forges, C.P. 500, Trois-Rivières (Québec) G9A 5H7, Canada; alencar.soares.bravo@uqtr.ca (A.B.); koffi@uqtr.ca (D.K.); 2 Laboratory of biomaterials, University of Quebec at Abitibi-Témiscamingue, 445, boul. de l'Université, Rouyn-Noranda (Quebec) J9X 5E4, Canada; fouad.erchiquui@uqat.ca

**Keywords:** biocomposites, green composites, damage modes, acoustic emission, fuzzy logic

## Abstract

Despite the knowledge gained in recent years regarding the use of acoustic emissions (AEs) in ecologically friendly, natural fiber-reinforced composites (including certain composites with bio-sourced matrices), there is still a knowledge gap in the understanding of the difference in damage behavior between green and biocomposites. Thus, this article investigates the behavior of two comparable green and biocomposites with tests that better reflect real-life applications, *i.e.*, load-unloading and creep testing, to determine the evolution of the damage process. Comparing the mechanical results with the AE, it can be concluded that the addition of a coupling agent (CA) markedly reduced the ratio of AE damage to mechanical damage. CA had an extremely beneficial effect on green composites because the Kaiser effect was dominant during cyclic testing. During the creep tests, the use of a CA also avoided the transition to new damaging phases in both composites. The long-term applications of PE green material must be chosen carefully because bio and green composites with similar properties exhibited different damage processes in tests such as cycling and creep that could not be previously understood using only monotonic testing.

## 1. Introduction

In “biocomposites”, natural fibers are used as an environmentally friendly alternative to fibers traditionally used in composites. The advantages of natural fibers include their low density, high specific strength, enhanced energy recovery, CO_2_ neutrality after burning, easy processing, bio-degradability and low cost [1,2]. The improvement of the green nature of composites with a natural fiber content of less than 50 wt% (*i.e*., with one or more thermoplastics as the major constituent of the matrix) can be achieved using a bio-sourced matrix and natural fibers. When the entire composite is bio-sourced, the term “green composite” is used.

Several studies have investigated the particular mechanical characteristics of biocomposites. Migneault *et al.* [3] studied the properties of an HDPE polymeric matrix impregnated with white birch fibers under various loads using a rheometer. The authors found that not only the mechanical properties but also the melting properties were affected by the fiber weight. Raj and Kokta [4] arrived at similar conclusions using aspen fibers. The use of a coupling agent (CA) is also commonly examined. Lu *et al.* [5] provided evidence that among the various CAs tested, maleated polyethylene (MAPE) was the best option. In addition, Colom *et al.* [6] demonstrated the “bridging” effect of MAPE using scanning electron microscopy (SEM). Adhikary *et al.* [7] investigated the differences in stability, mechanical properties and microstructure between recycled and virgin HDPE using fibers of *Pinus radiata*. The results were extremely encouraging from an ecological perspective because the results demonstrated that the mechanical properties of the biocomposite were the same regardless of whether virgin or recycled HDPE was used.

Due to compatibility issues of polymers and natural fibers, acoustic emission (AE) real-time monitoring is frequently used as a method to assess micro-damage mechanisms and their evolution in biocomposites [8]. Park *et al.* [9] evaluated the interface adhesion of various jute and hemp fiber-reinforced matrix composites using the micromechanical technique and AE. The results indicated that the CA content in the polypropylene (PP) blend increased the interfacial shear strength. The same was observed when natural fibers were treated with an alkaline solution. Akil *et al.* [10] analyzed AE during reloading (Felicity ratio), and the AE activity at low loads during unloading of pultruded jute/glass and kenaf/glass hybrid polyester composites and compared the results with kenaf fiber composites. Introducing a large amount of reinforcement appears to be extremely effective in jute fiber-reinforced laminates, whereas it did not yield comparable results in kenaf fiber laminates, which was attributed to the insufficient fiber impregnation. Santulli [11] characterized the damage due to low-velocity impacts on jute fiber-reinforced polyester composites using AE. Compared with observing damage under an optical microscope, AE is able to reliably measure the level of damage. Park *et al.* [12] investigated the interface and durability of alkaline and silane-treated jute fibers/PP composites by a micromechanical test combined with wettability and AE. The AE energy increased for the alkaline and silane-treated jute fibers/PP composites, whereas the AE energy for all three cases decreased distinctly after a boiling water test. Kobayashi [13] investigated the AE fatigue properties of a hemp fiber yarn-reinforced poly(lactic acid) composite (green composite). The results indicated that the unidirectional fibers in composites begin to split before final fracture, whereas matrix cracks and debonding between the matrix and fiber yarn occurred and accumulated stably in the textile composites.

A 100% ecological HDPE composite was first achieved by Braskem, a Brazilian petrochemical company (and America’s top producer of thermoplastics [14]) that developed a “natural (green) polyethylene” (NHDPE) sourced entirely from sugarcane. This was a technological breakthrough because, for the first time, two materials claiming to have identical mechanical properties but sourced differently could be compared in terms of their composite HDPE and NHDPE matrices. There is a knowledge gap in the understanding of the damage behavior of green and bio versions of composites with the same base formulation. In a preliminary study, which compared composites using these materials as a matrix, it was verified that the choice of a composite for a particular application must be judicious and should not only consider the mechanical properties [15]. Interestingly, it was revealed that even though the differently sourced composites exhibited similar static mechanical behavior, the internal damage evolution was completely different. Although this was a major breakthrough for analyzing damage modes in comparable bio and green composites, two important aspects of the damage process have not yet been studied.

The first of these overlooked aspects is cycling or the effect of a progressive accumulation of deformation during cyclic loadings. This process allows the mechanical verification of the changes in material properties, energy dissipation (hysteresis), plasticization and the correlation of the micro mechanism identified by AE with another mechanical indicator. This process is also important for documenting the material stability over cycling using measures, such as the Felicity effect, which uses the cyclizing response and AE. The second overlooked aspect regarding the differences between bio and green composites is how the internal damage mechanism evolves via cold flow or the tendency of a solid material to move slowly or deform permanently under the influence of mechanical stress. By maintaining a high level of constant stress on the specimen and analyzing the specimen, AE can verify the evolution of the deterioration mechanism in these two different composites. With this method it is also possible to compare a mechanical indicator evolution (strain) with AE response. Studies that investigate these aspects are important to expand the understanding of the degradation of these materials and to enhance the knowledge of their uses, such as in mechanical gears.

In this study, we investigate these knowledge gaps between comparable bio and green composites made from HDPE (or in the case of the green composite, NHDPE) for various fiber weights using creep and cyclic tests, which were not performed in previous work [15]. One of the most significant gaps in this past study was the fact that AE damage analysis was performed independently, *i.e.*, without a mechanical behavior equivalence. For this study, the damage model associated with the mechanical processes proposed in this study is based on the model introduced by Kachanov [16], which assumes that damage to a material can be interpreted in terms of the density of defects in that material. The damage mode characterization using of three known parameters (burst amplitude, counts and duration) for PE/birch is already known in the literature and it will be utilized in this article [17].

The previous study [15] just presented the quasi-static mechanical properties under simple monotonic testing and the AE of those testing. This article will explore much deeper the damaging process under different situations that will provide not only means of recording AE signal but an external mechanical indicator of damage for comparison of the actual damage evolution. In the case of cycle testing, this external measure is the change in Young’s modulus. For the creep testing, the external parameter is the strain evolution.

In sum, some essential elements of novelty of this article are: insights of the stress accumulation at the interface between fiber and matrix and detachment point using cyclic residual stress and modulus change; cyclic accumulation of AE; comparison of phases and modes in terms of mechanical and acoustic readings; comparison of damage measuring techniques; Felicity and Kaiser effect analysis; comparison between the differences of the mechanical phases and actual AE signals during creep; and damage surveillance using AE energy and AE hits number as criteria This paper is organized as follows. In Section 2, the materials, methods and procedures of the experimental testing are described. Section 3 presents and discusses the results. In this section, firstly the mechanical behavior and properties of the various composites, as determined from monotonic tensile testing, are discussed and compared. Secondly, the result of load-unload tests and the change in the mechanical behavior with cycling are discussed for the various materials. Third, an AE damage analysis based on cyclic testing is presented, and the results are discussed. Fourth, the differences between damage progressions for a fixed stress value are elucidated using AE. Fifth, the previous results are correlated with SEM images. Final conclusions are presented in Section 4.

## 2. Materials Preparations and Experimental Procedure

### 2.1. Materials

Industrial short fibers (thermomechanical pulping, 35 mesh size) of yellow birch (Betula alleghaniensis) were used in this experiment. The fibers were produced by the Lignocellulosic Materials Research Centre, Trois-Rivières (Canada) and dried at 60 °C in an air-circulating oven for 24 h before use.

The two thermoplastic matrices that were used were HDPE (Sclair^®^ 2909), donated by NOVA Chemicals, and NHDPE (SHA7260), donated by Braskem. MAPE (G2010), supplied by Eastman Chemical Company (Kingsport, TN, USA), was used as the CA. The maleic acid graft content was 1.5%, with a molecular weight of 15,000. The CA chemical composition leads to the formation of chemical bridges between the natural fibers and the HDPE matrix. The use of CA in quantities beyond 4 wt% can lead to self-entanglement among CA chains rather than entanglement with the polymer matrix [18]. For this reason, 3 wt% CA was used.

All specimens were prepared using a two-roll mill (Thermon C.W. Brabender, Model T-303) with a 0.6 gear ratio. The grains of the matrix were melted on rollers at 170 °C, and the fiber was subsequently added at the desired weight ratio (0, 10, 20, 30 or 40 wt%). Specimens were producing using a molding process at a temperature of 205 °C and using a hydraulic press for 20 min at a pressure of 10 MPa. Each specimen was weighed with a precision balance, and those with more than 1% void fraction were rejected. For each variation, a total of 10 samples were produced following this protocol. The first five were used for mechanical characterization and errors were reported. Once the quality of the samples was assured, the following were used to perform cycling (two samples) and creep (three samples) testing.

### 2.2. Experimental Testing

**Figure 1:**
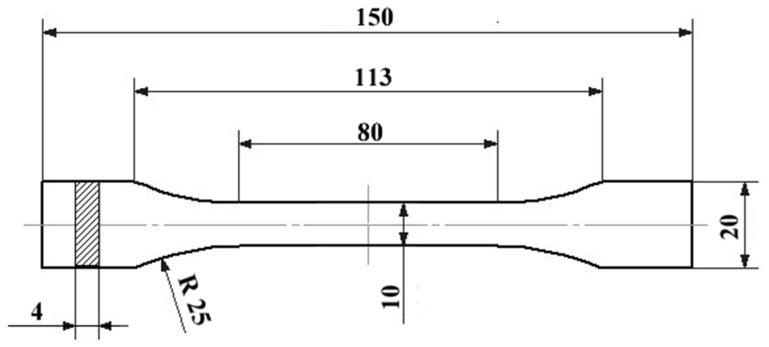
Specimens dimensions (in millimeters) according to the standards of ISO 527-4(1A).

**Figure 2:**
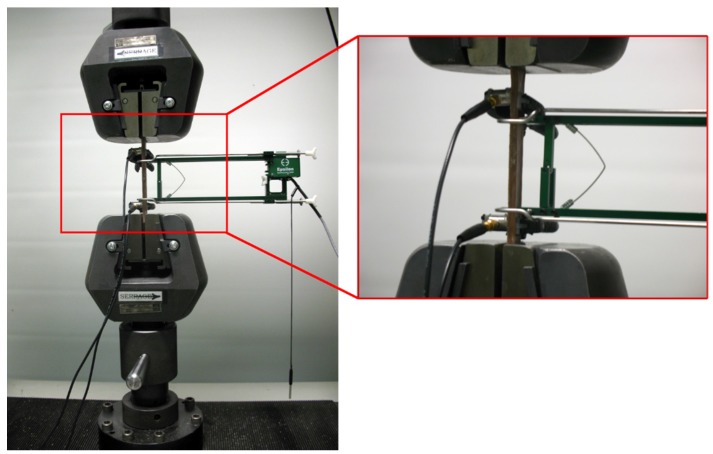
Tensile machine testing apparatus with acoustic sensors and extensometer on the testing sample.

At room temperature, monotonic and load-unload (R=min(σ)/max(σ)=0) tensile tests were performed following the standards of ISO 527-4(1A). The ISO norm requires that all residual forces induced in the specimen due to the clamping procedure must be removed before testing, whereas the equivalents ASTM D638/ASTM D3039 do not require this removal. The specimen dimensions are shown in Figure 1. The tests were carried out using an electromechanical testing machine, Instron model LM-U150, equipped with a 150-kN load cell (Figure 2). During tensile testing, a 25 mm extensometer was connected to the data acquisition system and fixed on the gauge length section of the specimen to record the strain evolution.

AE measurements were conducted using devices provided by Physical Acoustics Corporation (PAC), equipped with two PCI cards. Two sensors (type Micro-80 PAC, wideband 100–1000 kHz) were mounted to the surface of the test specimen at a spacing of 70 mm. An acoustic threshold level set at 35 dB was used to filter the background noise. A silicone adhesive gel was used as a coupling agent between the sensors and the specimen. Before each test, the quality of the coupling was verified using a Nielsen-Hsu pencil lead break.

The quality of the measured AE data depends mainly on the choice of the waveform system timing parameters, namely, the peak definition time (PDT), hit definition time (HDT) and hit lockout time (HLT). The employed values of these timing parameters are PDT = 40 µs, HDT = 80 µs and HLT = 200 µs [19].

## 3. Results and Discussion

### 3.1. Mechanical Properties

**Figure 3:**
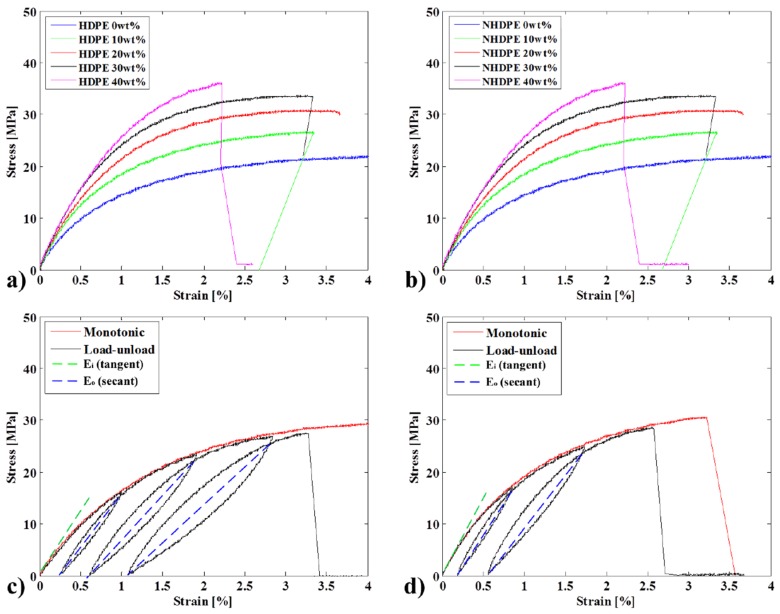
Tensile test results for specimens with different fiber weights for (**a**) HDPE; (**b**) NHDPE; (**c**) superimposition of the monotonic and load-unload test results for 20 wt% fiber HDPE and (**d**) NHDPE.

This section reviews the monotonic tensile properties and compares the applicability implications that the previous study failed to address [15]. Figure 3a and Figure 3b show the evolution of the stress-strain curves during monotonic tensile testing for an HDPE-based composite with no CA and for an NHDPE green composite with no CA, respectively. Both composites behave similarly with increasing fiber proportion, which reduces the ductility of the material while increasing the tangent Young's modulus (according to ISO-527) and the ultimate strength. Both composites blend well with the fibers, but as the fiber content increases above 30 wt%, they become saturated.

Figure 3c and d show an example of results for the 20 wt% fiber bio and green composite samples (respectively), with the monotonic and load-unload tests superimposed. 

The mechanical properties are summarized in Table 1. The manufacturing protocol used in this work shows good measurement reproducibility, with a low standard deviation. Considering the bio composite and the specimens without a CA, in increasing order of fiber content, the increase in the Young's modulus relative to the pure HDPE matrix specimens was 51.18, 71.65, 131.50 and 238.58%, and the increase in the maximum stress relative to the pure matrix specimens was 24.27, 35.41, 54.73 and 63.64%, respectively.

Adding a CA to an HDPE biocomposite has a minimal effect on the Young’s modulus, resulting in increases of only 4.72, −3.15, −3.94 and 14.17% for the specimens with fiber contents of 10, 20, 30 and 40 wt%, respectively. However, the CA improved the maximum stress, with respective increases of 2.00, 5.73, 12.91 and, remarkably, 41.41% compared with the corresponding specimens without a CA.

**Table 1:** Tensile test data: average ultimate strength and Young’s modulus [15].

Matrix Used (wt%)	Fiber Weight (wt%)	Coupling Agent (wt%)	Young’s Modulus (GPa)		Maximum Stress (MPa)		Strain at Failure	
			Mean	STD DEV	σmax (MPA)	STD DEV	d (%)	STD DEV
HDPE	0	-	1.27	0.07	22.00	0.86	-	
HDPE	10	0	1.92	0.19	27.34	1.99	3.63	0.64
HDPE		3	1.98	0.03	27.78	1.56	3.40	1.18
HDPE	20	0	2.18	0.19	29.79	1.47	5.07	0.68
HDPE		3	2.14	0.04	31.05	0.86	5.23	1.21
HDPE	30	0	2.94	0.13	34.04	1.13	3.50	0.39
HDPE		3	2.89	0.06	36.88	1.10	4.60	0.10
HDPE	40	0	4.30	0.34	36.01	3.03	1.74	1.01
HDPE		3	4.48	0.04	45.12	1.92	2.44	0.50
NHDPE	0	-	1.17	0.17	21.39	0.34	7.80	0.51
NHDPE	10	0	1.93	0.16	26.89	1.03	4.00	0.94
NHDPE		3	1.99	0.18	27.21	0.55	3.19	0.31
NHDPE	20	0	2.43	0.17	29.67	1.62	3.70	0.39
NHDPE		3	2.33	0.06	29.92	1.02	2.89	0.38
NHDPE	30	0	3.04	0.29	33.47	3.13	2.18	0.77
NHDPE		3	3.50	0.13	40.60	1.37	2.50	0.08
NHDPE	40	0	3.86	0.07	32.68	0.40	1.78	0.40
NHDPE		3	4.57	0.20	46.45	1.74	2.70	0.34

The NHDPE matrix was found to perform well; exhibiting markedly higher mechanical properties as the fiber content was increased (Figure 3b). Compared with the pure matrix, the samples without a CA and with fiber contents of 10, 20, 30 and 40 wt% exhibited Young's modulus improvements of 64.96%, 107.69%, 159.83% and 229.91% and maximum strength improvements of 26.89%, 38.71%, 56.47% and 52.78%, respectively.

The coupling agent is more effective in combination with the NHDPE and high fiber content. This effect can be seen with fiber weights of 30% and 40%, which increased the Young’s modulus by 36.22% and 55.91%, respectively. The same effect is observed in the maximum strength with similar improvements: 32.41% and 62.59% for fiber weights of 30% and 40%, respectively.

It is possible to verify other advantages of increasing the fiber ratio in NHDPE instead of HDPE: the application range increases due to the increase in the mechanical properties, and there is a financial benefit of using natural fibers (which can be obtained from waste). However, in this case, the use of CA is strongly recommended because it has a very beneficial effect on the properties of high-fiber green composites.

**Figure 4:**
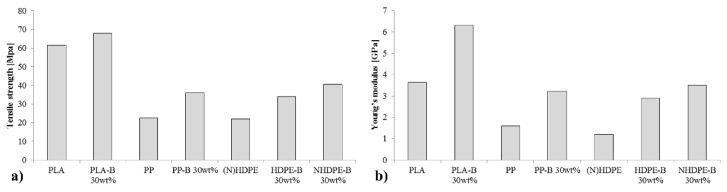
Comparison of the family of bio and green PE/birch composites developed with commonly used engineering plastics and composites [20,21,22]: (**a**) Young’s modulus (**b**) Tensile strength.

Comparisons of newly developed composites with those currently used for particular applications are particularly important. In this case, our goal is to replace plastic gears with an equivalent ecological solution. Plastic gears are often made with engineering plastics, *i.e.*, plastics that have a superior Young’s modulus and tensile strength that can transfer the load and maintain accuracy during gear meshing. The most used engineering plastics in gears are nylon (PA66) and acetal (POM) [20,22,23].

Reinforced polymers with synthetic fibers are also commonly used for gears when engineering plastics are unavailable or are too expensive. Commonly used reinforcements are short glass fibers (PP-SGF) and short carbon fibers (PP-SCF) [20,21].

For comparison, POM, PA66, PP, PP-SCF with 25 wt% and PP-SGF 25 wt% are compared with the new set of proposed composites (Figure 4). In this graph, both oil-based and natural HDPE occupy the same bar using the average value, which can be assumed because their differences in these cases are minimal. The difference in the Young’s modulus is 0.10 and 0.09 GPa for the pure polymer and the 40 wt% fiber-reinforced polymer with CA, respectively, and the difference in the maximum strength is 0.61 and 1.45 MPa, respectively.

The original HDPEs without fibers have a low Young’s modulus and tensile strength for this application. The Young’s modulus of POM is 166.7% greater than that of pure (N)HDPE and that of PA66 is 158.3% greater. With the use of 40 wt% of fiber and CA, a stiffness that is comparable with the commonly used engineering plastics for gears can be obtained. The difference in the tensile strength is 41.3% greater than POM and 78.3% greater than PA66 compared with that of the (N)HDPE 40 wt% birch fiber with CA. In terms of the Young’s modulus, the proposed material is increased by 28.9% and 31.1% compared with that of POM and PA66, respectively. Thus, the proposed material appears to have advantages over engineering plastics. Nevertheless, further investigation of the NHDPE behavior and internal structure is necessary because this is an extremely recently discovered material.

Pure PP has relatively slightly better properties compared with those of pure (N)HDPE. The Young’s modulus of PP is 41.7% greater than that of (N)HDPE, and the tensile strength is 72.7% greater. Interestingly, when synthetic fibers are used, the mechanical properties increase much more than that with the addition of natural fibers in the (N)HDPE. PP-SCF with 25. wt% has a tensile strength that is 28.3% greater than that of (N)HDPE with 40 wt% and a Young’s modulus that is 233.3% greater.

Regarding the tensile strength, the difference is 1.96 and 2.73 greater for the same materials, respectively. PP has the most similar properties compared with those of HDPEs. The Young’s modulus is 41.7% greater and the tensile strength is 72.7% greater compared with those of pure HDPE. PP-SCF with 25 wt% has a tensile strength that is 28.3% greater than the (N)HDPE with 40 wt%, and PP-SGF with 25 wt% is greater by only 8.7%. In the case of the Young’s modulus, the difference is larger. The Young’s modulus of PP-SCF with 25 wt% is 233.3% greater, whereas that of PP-SGF with 25 wt% is 66.7% greater. Natural fibers cannot increase the stiffness at the same rate as synthetic fibers do. However, their use can lead to a reasonable maximum tensile strength and surpass the Young’s moduli of engineering plastics. These are extremely interesting properties, and the introduction of the NHDPE matrix should not be an excessively complicated or costly task because many industries already work with HDPEs.

As discussed above, natural fibers have several important aspects compared with other fibers. Fibers obtained from plants have cellular structures that are assembled through a hierarchical process in nature and, therefore, are neither homogeneous nor chemically compatible with ordinary polymers [24]. Thus, the study of the internal micro-phenomena and degradation processes after being submitted to various stress scenarios are important, particularly to highlight the differences between HDPE and NHDPE in terms of fiber behavior.

### 3.2. Property Degradation and Failure

Basic material mechanical properties, such as strength and ductility, can be obtained from simple monotonic tensile tests. However, the stress-strain behavior determined from simple monotonic tensile tests can be quite different from that obtained under cyclic loading. Because no standard test method exists for load/unload tests, a sufficient number of cycles was chosen to cover the different phases that describe the behavior of the biocomposite. At room temperature, load-unload longitudinal tensile tests were performed as follows. First, loading was applied to the specimen until a certain strain was achieved, after which the loading was removed. The material was then subjected to a higher strain level.

As Figure 3c,d show, the experimental cyclic test superposed very well the corresponding monotonic curve. Mechanically, this indicates that the composite structure is not subject to cyclic hardening or softening, which would result in internal structural deformation. The composites do not show any significant relationship between the accumulation of plastic deformation and the maximum stress, even in the later cycles.

Conversely, significant hysteresis was seen under cyclic loading and unloading. This phase lag leads to a dissipation of mechanical energy [25]. For each cycle, the unloading and reloading curves deviated from the line connecting the unloading crossing point with the loading line and the zero loading (fully unloaded) point. This line represents the secant to the curves, and its slope defines the effective or mean Young’s modulus, which is generally not equal to the initial Young’s modulus [26].

The change in the effective elastic modulus (secant) is commonly used to document irreversible changes in the properties of materials due to the application of cyclic stresses or strains. The cumulative damage, *D_m_* (mechanical damage index), is defined as follows:

(1) $$D_{m}=1-E_{i}/E_{o}$$

where *E_i_* is the Young’s modulus after the ith cycle and E_o_ is the initial modulus.

**Figure 5:**
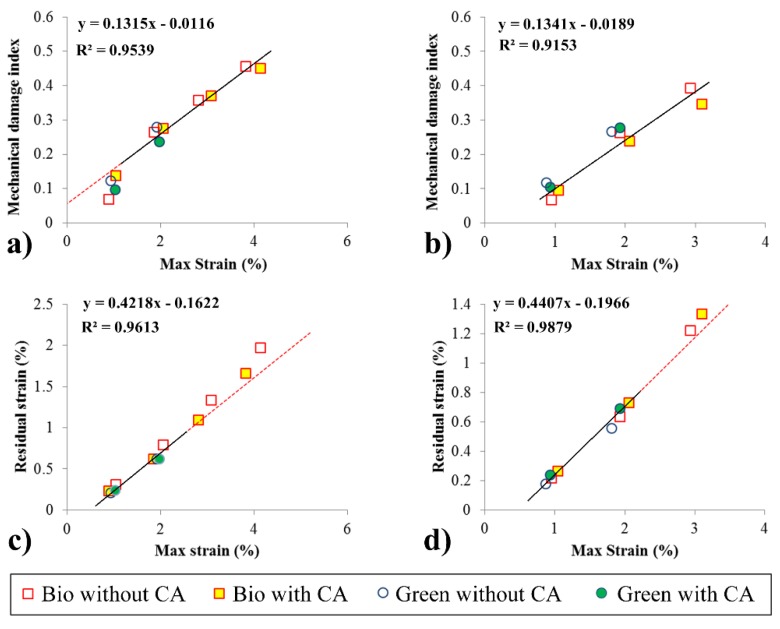
Mechanical damage index *versus* strain for 10 wt% (**a**) and 30 wt% (**b**) fiber composites. Residual strain *versus* strain for 10 wt% (**c**) and 30 wt% (**d**) fiber composites.

Figure 5a,b presents the relationship between the damage accumulation and maximum strain for a cycle for all specimens of 10 and 30 wt% fiber, respectively. For specimens with low fiber content (10 wt%), there are two distinguishable phases. Damage accumulates differently in the first two cycles. This initial lower degradation is due to the high elasticity present at 10 wt%, corresponding to only weak low plastic effects on the material. A second linear phase was only present in the biocomposites, as the green composites failed earlier. Considering the third, fourth and fifth cycles, a linear slope of 0.1315 /% (increase in D by increase of maximum strain) can be obtained, with a coefficient of determination (R^2^) of 95.39%.

The traditional polymer suffered a rupture around D = 0.45, independent of the use of CA. In the natural material, this rupture occurred at D = 0.25, which was also independent of the CA. Interestingly, as the fiber continent increases, the differences in behavior for the two types of composite decrease. At 30 wt% fiber, the green composites still fail at D = 0.25, although the biocomposite fails around D = 0.35. The viscoelastic effects in this case are less marked, as there is no visible viscoelastic phase. This change in behavior can be attributed to the high fiber content in the composite, as the wood fiber exhibits very low viscoelastic behavior [24]. Interestingly, compared to the 10 wt% specimens, the slope of the damage index remains the same for the 30 wt%, 0.1341, with an R^2^ of 91.53%.

Figure 5c and d show the evolution of residual strain for each cycle at different fiber weights. The abscissa shows the maximum strain obtained in each cycle before the unloading phase begins.

These images show that, in the first few cycles, the composites behave as a homogeneous material, with the fibers and matrix experiencing the same strain. Above a certain limit, due to the differences in their Young’s moduli, shear forces accumulates in the matrix–fiber interface. Consequently, the matrix will be compressed, and the fiber will experience tensile stress after the unloading phase. The shear stress in the components will increase, ultimately resulting in the detachment of the fibers from the matrix. If this detachment occurs, the matrix is freed from the fibers resulting in increased residual strain [17]. Due to the similar mechanical properties of HDPE and NHDPE, this phenomenon occurred at the same maximum strain, approximately 2.5%, independent of the matrix used or the fiber ratio. Nevertheless, the precise effect of the CA on the damaging process must be determined by supplementing these mechanical analyses with AE.

### 3.3. Mode Detection

Several modes of micro failure are found in composites. For this reason, knowing the mechanical properties and general degradation is not sufficient to completely understand the damage modes. The changes in mechanical properties are often related to changes in the damage modes. AE tests were performed to examine the microstructural failure events contributing to the behaviors of different composites.

Damage-mechanism analysis has traditionally been performed using the simple discrimination of mode by hit amplitude approach [27,28]. However, this methodology can be inaccurate for complex materials [29], especially biological materials (*i.e*., cellular structures assembled through a hierarchical process in nature) using thermoplastics (with strong damping properties) [17,30]. In this case, a more complex analysis is recommended. Specifically, fuzzy logic systems are recommended [31,32], which can detect clusters in the data even when the boundaries between groups overlap [33].

The AS for damage-mode identification in the PE/birch composite family using three known parameters (burst amplitude, counts and duration) is documented in the literature [15,17] and is used in this study for all composites. Table 2 shows the AS values. These values are independent of the fiber weight used and are valid for HDPE and NHDPE composites with short birch fibers [15].

**Table 2:** Summary of the damage acoustical signature [17].

Damage Mode	Matrix Micro-Cracking	Matrix/Matrix Friction	Decohesion	Matrix/Fiber Friction
Amplitude (dB)	35–45	40–55	45–60	55–85
Duration (μs)	1–80	20–120	50–200	100–600
Counts	1–10	8–20	16–35	30–120

**Figure 6:**
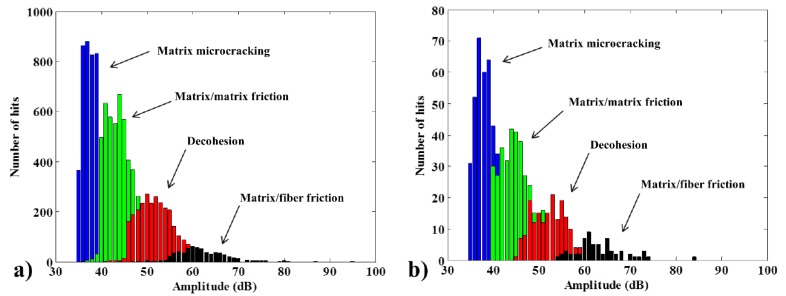
Typical amplitude histogram successfully discriminated by fuzzy logic for bio (**a**) and green (**b**) composites at 10 wt% fiber with no coupling agent.

The distribution of the AE amplitude takes the form of groups, which may overlap, reflecting the failure mechanisms. The results of amplitude histograms for 10 wt% fiber bio and green composites are documented in Figure 6.

The histograms show that, at the end of the test, both composites have similar distributions. Matrix microcracking (blue) is most common, followed by matrix/matrix friction (green). The red group (decohesion) is usually an indicator of the adhesion quality between the fiber and the matrix. In this case, both composites have a similar AE indicator for fiber adhesion quality, which indicates that the green matrix absorbs the short natural fibers with the same adhesion quality as the normal matrix. The similar proportions of matrix/fiber friction also corroborate this hypothesis. Interestingly, both composites have similar histograms, and the green matrix in particular is not very AE-responsive; for a similar level of stress, it normally emits 10-fold less AE signals (Figure 6).

### 3.4. Cyclic AE Burst Intensity and Mode Analysis for Cyclic Testing

Once the mechanically damaging overall behavior of the composites was determined, verification of the evolution of the internal micro-events was of interest. The changes in the mechanical properties are often related to changes in the damage modes. For example, decohesion usually leads to a decrease in composite strength, but a decrease in strength is not always associated with poor adhesion and decohesion [34]. For this reason, knowing the mechanical properties and general degradation is not sufficient to completely understand the damage modes.

**Figure 7:**
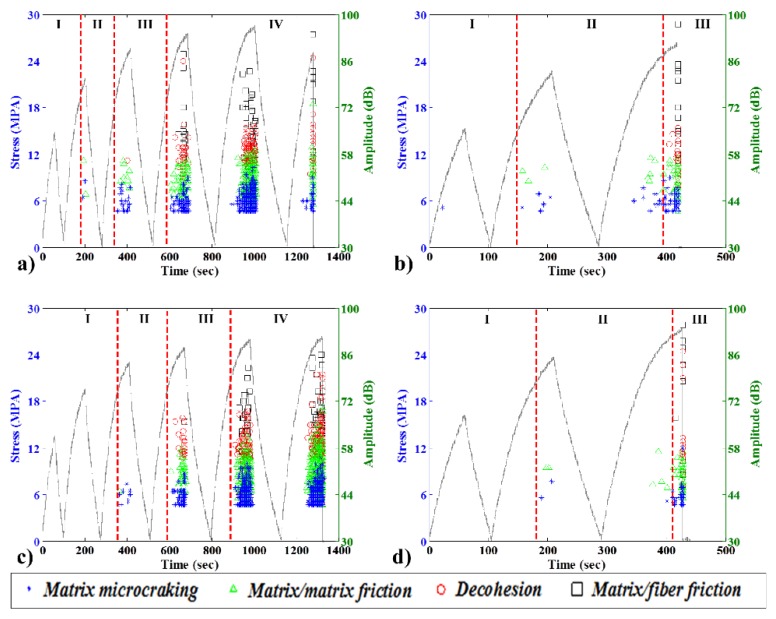
Typical load-unload stress curves (left vertical axis) and burst amplitudes with damage mode classification (right vertical axis) for 10 wt% specimens: (**a**) bio, no CA; (**b**) green, no CA; (**c**) bio with CA; and (**d**) green with CA.

Figure 7 shows the stress curve and the AE event modes and amplitudes over time for 10 wt% specimens. This fiber content was chosen because the specimen ruptures occurred at higher strains, resulting in more AE mode events. In this graph, the blue points correspond to matrix microcracking, green triangles correspond to matrix/matrix friction, red circles correspond to decohesion between the fibers and the matrix, and black squares correspond to matrix/fiber friction.

The data for the HDPE biocomposite without a CA exhibit four phases. The first phase is elastic, without significant AE events. The second is characterized by the onset of matrix microcracking and matrix/matrix friction events. The third has a rapid increase in the AE event rate and the first appearance of decohesion. The fourth has a very high rate of AE activity and all damage modes, including matrix/friction.

Upon the addition of a CA, however, the data reveal a different damage behavior. After phase 1, during which no AE events were recorded, phase 2 began with the first AE events. In contrast, in the specimen without the CA, the first significant acoustic events are only matrix microcracking at similar levels of stress for both specimens. The third phase featured a few bursts of decohesion and matrix/fiber friction, the last of which had low amplitude. Finally, phase four had the same behavior as the specimen without CA but started much closer to the specimen total failure.

In the case of the green composite, the data reveal completely different behavior. With no CA, three phases were observed. After the first phase, during which no significant AE events were recorded, the second phase began with a few low-amplitude events (primarily microcracking and matrix/matrix friction). Phase 3 contained many high-amplitude bursts (decohesion and matrix/fiber friction), resulting in fragile breakage. In general, there were few AE hits, because even at a fiber content of 10 wt%, the specimen ruptured at a low strain value. Upon the addition of 3 wt% CA there was no decohesion in phase 2; rather, the decohesion started in phase 3. This finding might be related to the fact that the specimens were made more resistant by the addition of a CA. 

Apart from the completely different damage process, however, there was only a small difference in the mechanical properties of the HDPE and NHDPE composites. The NHDPE green composites were found to be much more fragile and more likely to suffer sudden complete failure than the HDPE.

The low burst number is still an indication that the material was very fragile. This finding indicates that the choice of a composite for a particular application must be judicious and should take into account not only the mechanical properties but also the damage processes of the composite. 

### 3.5. Cyclic AE Energy Sharing and Correlation with Mechanical Results

Once the AS is defined, the quantitative mode participation in specimen degradation can be assessed. To evaluate the extent of the damage caused by each mode and its contribution to the overall failure, we used the damage energy participation index *D*(*AE*), defined as follows:

(2) $$D{\left(AE\right)}_{i}=\frac{E_{i}}{\sum_{\text{i}=1}^{4}E_{i}}$$

where *E_i_* is the cumulated damage energy of mode *i*.

**Figure 8:**
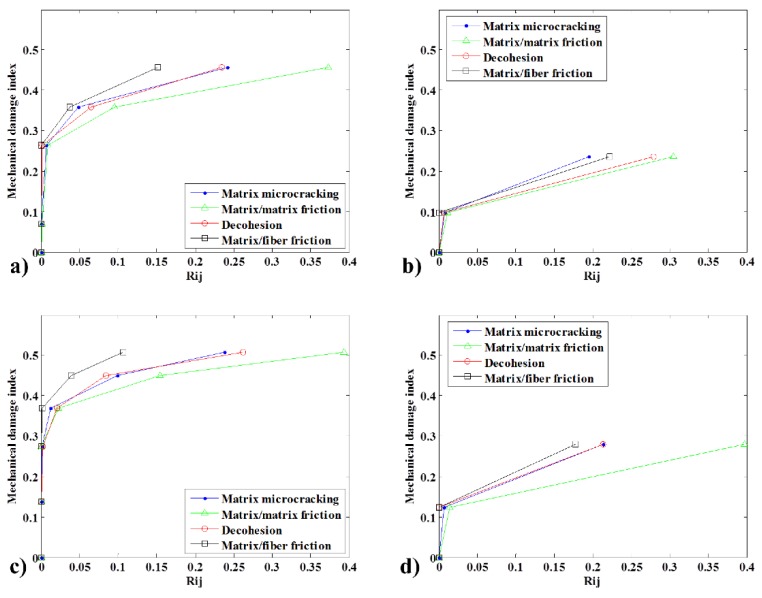
Comparison of the specimen typical damage using the mechanical damage index and cumulative AE damage measured using energy by mode for 10 wt% fiber specimens: (**a**) bio, no CA; (**b**) green, no CA; (**c**) bio with CA; and (**d**) green with CA.

It is interesting to correlate the AE analysis with the mechanical behavior. Figure 8 is useful because it permits a comparison of the measured mechanical and AE damages. For the 10 wt% specimen bio-composite without CA, significant AE damage was observed only at 35.83% mechanical damage, after three cycles. At this point, the mechanism of matrix/matrix friction contributed to 9.56% of the damage. Decohesion and microcracking accounted for similar proportions, 6.51% and 4.82%, respectively. Matrix/fiber friction was the least important damage mode, with a contribution of 3.71%, resulting in a total contribution of 24.61% to the measured specimen AE damage.

As documented in Figure 8a, at final failure, the specimen had a mechanical damage index of 45.63%. According to the AE energy, the contribution of each mechanism to the failure was as follows: matrix/matrix friction 37.26%, matrix/microcracking 24.15%, decohesion 23.43% and matrix/fiber friction 15.16%.

As shown in Figure 5, the matrix and fiber of the material uncoupled started at 4.2% residual strain or 0.28 of mechanical damage. Figure 8a shows that, just after this cycle, a substantial amount of AE activity occurred.

In the case of the biocomposite with CA, the first considerable damage occurred at 27.47% of the mechanical damage. At this stage, matrix/matrix friction accounted for 15.48% of the damage, matrix microcracking for 9.91% and decohesion for 8.41%. Matrix/fiber friction had a marginal contribution of 0.16%. At failure, the mechanical index was 50.83%. The material could support 5.20% more mechanical damage upon the addition of CA. The mode that contributed most to the failure energy was matrix/matrix friction with 39.34%, followed by matrix microcracking at 26.22%. Although the value of decohesion remained stable at 23.80%, there was a decrease in matrix/friction participation to 10.65% (4.51% less). This indicates that fewer free fibers were available in the composite to cause this mode.

In the case of the green composites, the first significant AE result was seen at 9.68% mechanical damage. At this point, matrix/matrix friction had contributed 1.15% of the damage, matrix microcracking 0.78% and decohesion 0.70%. Matrix/fiber friction had not yet damaged the material, and the total AE measured damage was 2.63%. At failure, with 23.61% mechanical damage, the energy mode shares were 19.51%, 30.43%, 27.86% and 22.20% for matrix microcracking, matrix/matrix friction, decohesion and matrix/friction, respectively.

In the case of the green composite with CA, the first noticeable AE damage was recorded with 12.34% mechanical damage at an AE damage of 2.05%. In this case, there was no evidence of decohesion or matrix/fiber friction. By the end, the matrix/matrix friction was again the mode that generated the most energy, contributing 39.65%. The effect of the CA was noticeable in terms of decohesion. However, it was the third, not second, most damaging mode, with a contribution of 21.28% compared to 21.30% for matrix microcracking. Matrix/fiber friction contributed 17.77% of the AE damage.

Overall, the addition of CA stabilizes the composite structures, as evidenced by the fact that those with CA have a higher level of mechanical damage prior to failure.

More interestingly, the CA has a substantial effect on the perception of AE damage compared to the mechanical damage. If we compare the aforementioned cases in which a representative AE was first observed and the ratio of AE energy damage by mechanical damage at those points, we see that, for the case of the biocomposite without CA, the AE damage represented 68.69% of the corresponding mechanical value. With the addition of CA, this value drops to 15.70%. The same behavior is seen in the green composite; without CA, at the first representative AE value, the AE damage index was 27.18% of the mechanical counterpart. When CA was added, this value dropped to 16.64%.

### 3.6. Cyclic Felicity and Kaiser Effects

The stability of a composite is also indicated by the presence of Kaiser or Felicity effects. The Kaiser effect [35] occurs when, in the cyclic AE test, the first activity is only seen when the constraints previously exerted on a material are reached. The Felicity effect [36], a term used when there is no Kaiser effect, can be described as the occurrence of events in AE before the previously applied maximum stress is reached. This is an undesirable state from a damage point of view, as it indicates that rapid degradation of material properties has occurred.

**Figure 9:**
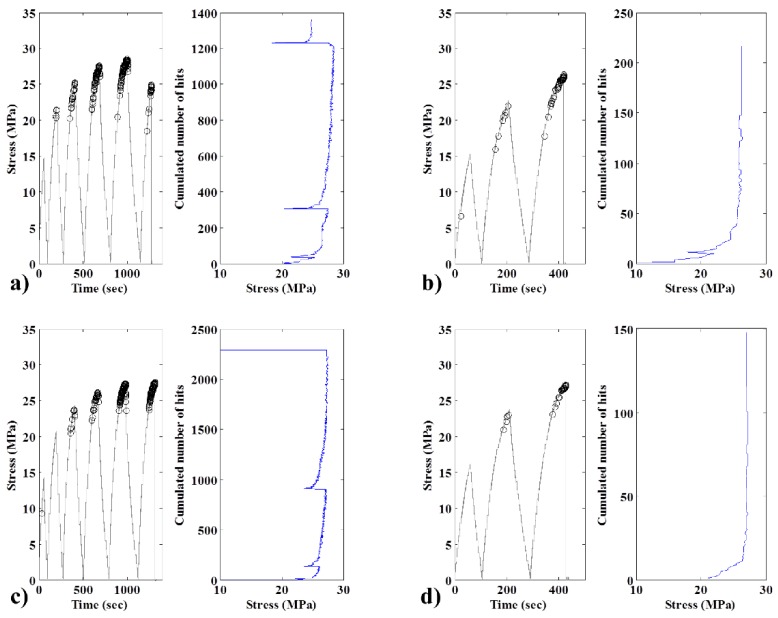
Typical Kaiser and Felicity diagrams for 10 wt% fiber specimens: (**a**) bio, no CA; (**b**) green, no CA; (**c**) bio with CA; and (**d**) green with CA.

According to Pollock [37], insignificant defects tend to indicate the Kaiser effect, whereas structural defects tend to represent the Felicity effect. In Figure 9 (left), the stress/strain curves feature acoustic events, which are represented by circular markers. These results can be confirmed by the acoustic measurements presented to the right or by tracing the evolution of the cumulated number of bursts with stress.

We note the presence of a Felicity effect for both biocomposites after the first cycles. Nevertheless, the addition of CA decreased not the occurrence but the intensity of the Felicity effect. In both cases, this effect increases with the number of cycles.

In the case of green composites, the addition of CA had a very beneficial effect. In this case, only the Kaiser effect was observed. In contrast, in the absence of CA, only the first cycle had a Kaiser effect, as the degradation of the material had not completely started. Beginning with the second cycle, we observe the presence of the Felicity effect. 

Ultimately, the occurrence of the Felicity effect in the material accelerates the degradation. This degradation can lead to a final break before the material reaches the theoretical limit of maximum elongation (as seen in Figure 5c,d for specimens without CA). As we note that the addition of CA tends to weaken the Felicity effect, this indicates that specimens without CA are more prone to break prematurely in practical applications.

### 3.7. Creep Mode Sharing Analysis

**Figure 10:**
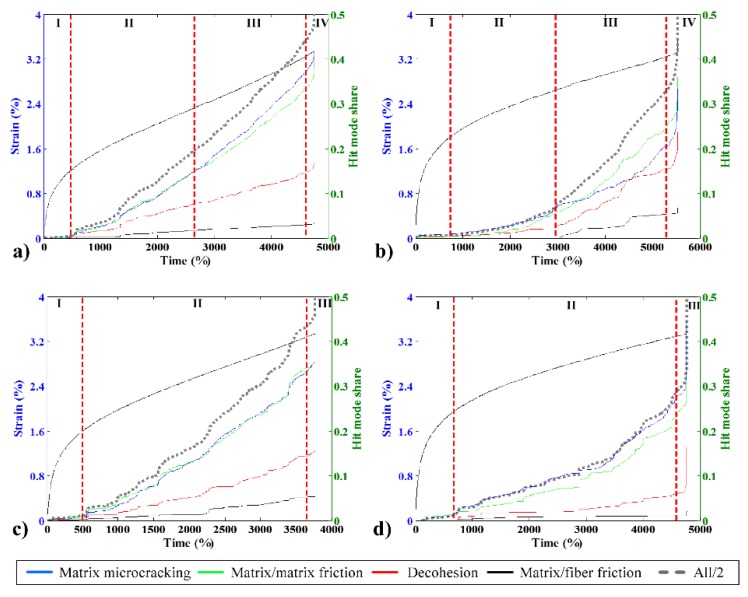
Typical creep strain curves (left vertical axis) and hit mode share participation (right vertical axis) for 10 wt% fiber specimens: (**a**) bio, no CA; (**b**) green, no CA; (**c**) bio with CA; and (**d**) green with CA.

To further analyze the damage processes of the composites, it is important to verify the damage evolution using creep tests. Creep is the slow deformation of a material under a constant level of stress and is thus very important for long-term structural applications. To accelerate the appearance of damage, the creep tests were performed using 80% of the quasi-static limit strength. In this particular case, we expect to obtain small values from the very beginning of the test. Nevertheless, it is possible to verify three very distinct phases with regards to the AE activity in every test, as indicated in Figure 10. The maximum crosshead speed was 1 mm/min.

To understand the mode influence over the degradation process, we will use the damage participation ratio (*R_i_*) proposed by Gong *et al.* [38], which is defined as follows:

(3) $$R_{i}=\frac{N_{i}}{\sum_{i=1}^{4}N_{i}}$$

where *i* refers to the different modes of damage and *N* refers to the cumulated number of bursts for one failure mode. Using this definition, the participation of each damage mechanism as a function of time for the creep test is shown in Figure 10.

For this test, we use the composite with a lower fiber ratio (10 wt%) because it allows for a higher strain at failure and thus activates more AE events before the rupture.

Using this definition, it is possible to verify how the burst mode participation of each mode evolves over time. The sum of all four modes is also represented in this figure by a dashed gray line; however, in this case, the value shown on the right side scale must be doubled, giving the rupture point a value of one. Regarding the AE activity, all of the composites without CA have four distinct phases. In the first phase, which includes the elastic linear behavior and the initial plastic behavior where the strain curve is non-linear, AE activity remains very low. The detection of the first significant AE indicates the beginning of the second phase. Thenceforth, the AE energy increased constantly, indicating intense plastic deformation with a constant increase in the strain value, the second phase. In the middle of linear strain increase phase, the third phase starts as the proportion of AE mode share of matrix related modes began to change. The fourth phase was characterized by a rapid increase in energy prior to final failure.

In the case of the bio composite, matrix microcracking and matrix/matrix friction had the same share of hits, but the distance between the two began to increase in the begging of the third phase (around 2800s), as matrix microcracking became more predominant until final failure (41.34% over 37.63%). In the case of the green composite, the same phenomenon was observed, and matrix microcracking and matrix/matrix friction had roughly the same hit share during the second phase; the difference between the two started increasing until the final failure from the second half of the linear strain increase zone (around 3300 s). In this case, matrix/matrix friction became predominant (35.93% over 32.28% of matrix microcracking).

The use of a CA avoids the transition to the third phase in both cases, as the as share between matrix microcracking and matrix/matrix friction remains the same throughout the testing. This change in AE activity means that the CA prevented the initiation of an extra internal damage mechanism, probably because the matrix and fiber are better linked and internal dislocations are prevented. This hypothesis is reinforced by the fact that, in both cases, decohesion decreased, meaning that the CA was effective, especially in the case of the green composite.

These results are extremely interesting compared with results from the literature. In a previous study [15], it was revealed that one of the major drawbacks of NHDPE application compared with HDPE was the fact that, with standard tensile testing, it was impossible to detect any AE activity until being extremely close to final failure. Nevertheless, the creep testing showed that it is possible to detect AE activity in this case, even if the acoustic behavior is much different from that of HDPE with fewer events. Thus, in long-term applications of this material, AE equipment could be used to verify the structured aging with regards to creep to predict when replacement is required and to avoid catastrophic failures.

### 3.8. Creep AE Burst Intensity and Mode Analysis

**Figure 11:**
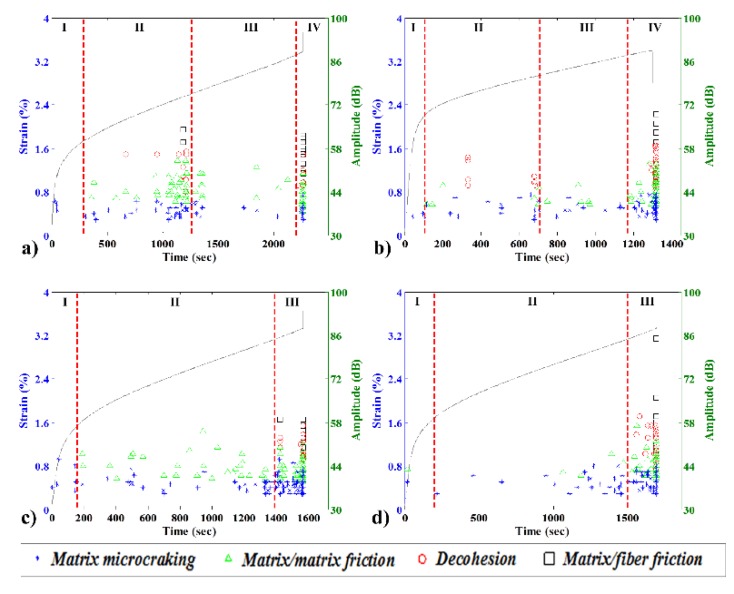
Typical creep strain curves (left vertical axis) and burst amplitudes with damage mode classification (right vertical axis) for 40 wt% specimens: (**a**) bio, no CA; (**b**) green, no CA; (**c**) bio with CA; and (**d**) green with CA.

The evolution of the strain and the AE event mode and amplitude over time for 40 wt% bio and green composites specimens with and without CA is shown in Figure 11.

The first phase of every test was characterized by the initial non-linearity of the strain evolution, with a few bursts of matrix microcracking that were independent of the composite used. As the basic stress value was highly elevated, the AE data indicates that little internal movement in the material was achieved by the matrix cracking while the force was being applied to the specimen. For all the composites analyzed in this study, the beginning of the second linear phase was marked by a particular AE event.

In the case of a bio composite without CA, the second phase started with the onset of matrix/matrix friction events. In the second phase, this composite presented moderate to elevated levels of AE activity. The activity continued increased constantly until a maximum in middle of the linear strain increase. At this, maximum bursts of all modes could be seen. In the following third phase, there is no changes in the material strain increase rate. Nevertheless, the AE activity remained calm for this third phase. The fourth and last phase is marked by a sudden increase in the AE activity of all modes.

The effect of CA was clear in the creep testing, as during the entire second phase, this composite presented moderate levels of AE activity of matrix microcracking and matrix/matrix friction. The CA had a clear effect, as no decohesion of fibers was observed in the second phase of this particular test.

As expected, the green composite without CA had fewer AE events than its bio counterpart. The second phase started with matrix microcracking and a matrix/matrix friction event. Additionally, some decohesion was found during the second phase. In this composite, there was also a third phase in the linear strain increase period. This behavior is very similar to that of the equivalent biocomposite, but with lower AE activity. The third phase has a lower AE activity and remarkably no decohesion bursts.

The third phase can be described as the onset of a chain mechanism that rapidly degrades the composite structure and leads to ultimate failure. This phase is characterized by a rapid increase in the AE activity rate and the level of all modes prior to failure.

Interestingly, both types of composites without CA at 10% or 40% wt% fiber present a change in the AE emission behavior in the middle of the linear strain increase zone. There are also similarities for the bio and green composites with CA. Both have lower AE activity, and neither show any decohesion in the second phase of this particular test.

### 3.9. Fractured-Surface SEM Images

**Figure 12:**
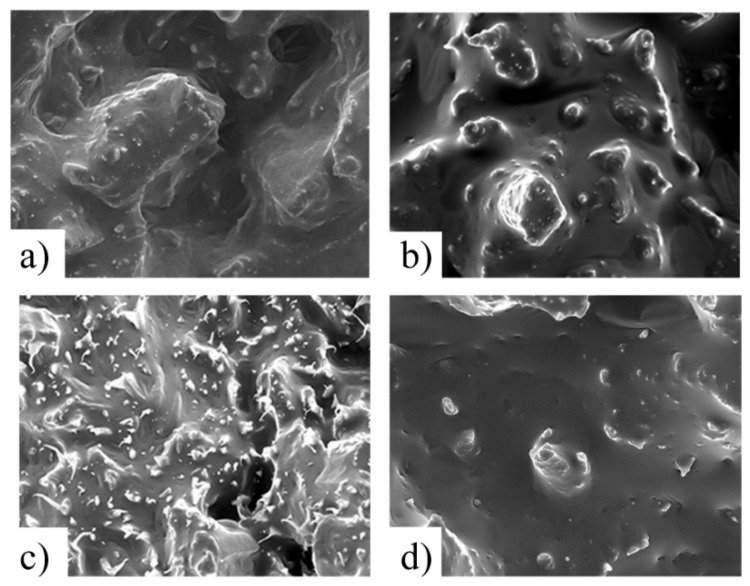
SEM images of the fractured face for 40 wt% specimens (at 500× magnification): (**a**) bio, no CA; (**b**) bio with CA; (**c**) green, no CA; and (**d**) green with CA.

Images of a fractured surface of metalized specimens obtained using SEM (JSM 5500, Jeol, Japan) at an acceleration voltage of 15 kV. This images can also reveal the primary cause of failure. In this section, we present the results for specimens used in the previous section (40 wt% fiber) at a magnification of 500× (Figure 12).

In the first column, we compared biocomposite samples with and without CA. Figure 10a shows the state of the surface of the HDPE-based composite without CA. A large amount of deformed material and fiber holes is observed. Comparing the green composite equivalent (Figure 12c), we see that the fiber holes are still present, but the matrix has less deformation, indicating a more brittle fracture. The relative low deformation might explain why much less AE activity is seen in the green composites, *i.e.*, there are fewer modes like matrix microcracking and matrix/matrix friction.

In both cases, the use of CA improved the fiber/matrix adhesion (*c.f*., Figure 12b,d), which reduced decohesion, as verified by the AE results. Nevertheless, the green composite surface was less deformed, which contributed to the lower activity, which in turn corroborated the AE results.

## 4. Conclusions

In this paper, we conducted a comparison of bio and green composites based on an HDPE matrix with birch fiber using monotonic tensile, cyclic and creep tests. Tests were conducted to investigate the mechanical properties, evolution and damage mechanisms using AE testing. Green composites are the most ecologically friendly solution. Coupled with a high fiber ratio and the use of a CA, the application range can be broadened due to the improved mechanical properties, with the financial benefit of using natural fibers.

Experimental cyclic test superimposed very well the corresponding monotonic curve. Mechanically, this indicates that the composite structure is not subject to cyclic hardening or softening, which would result in internal structural deformation.

In the first few cycles, the composites behave as a homogeneous material, with the fibers and matrix experiencing the same strain. Above a certain limit, due to the differences in their Young’s moduli, shear forces accumulate in the matrix–fiber interface. Due to the similar mechanical properties of HDPE and NHDPE, this phenomenon occurred at the same maximum strain, approximately 2.5%, independent of the matrix used or the fiber ratio.

After imparting mechanical damage, the evolution of the internal micro events was observed using AE. The damage modes were identified using a known fuzzy algorithm. Comparing the mechanical results with the AE, the addition of CA markedly reduced the ratio of AE damage to mechanical damage. Also, during the load-unload tests, the NHDPE green composites were found to be much more fragile and more likely to suffer sudden, complete failure than the HDPE. The low burst number is still an indication that the material was very fragile.

The addition of CA in the biocomposite decreased the intensity of the Felicity effect but not the occurrence of it. In the case of green composites, the addition of CA had an extremely beneficial effect because the Kaiser effect was dominant. Overall, the addition of CA stabilizes the composite structures, as evidenced by the fact that composites with a CA had a higher level of mechanical damage prior to failure and showed fewer decohesion events.

During the creep tests, the use of a CA avoided the transition to new damaging phases in both composites, as the share between matrix microcracking and matrix/matrix friction remains the same throughout the testing. This change in AE activity means that the CA prevented the initiation of an extra internal damage mechanism, probably because the matrix and fiber are better linked and internal dislocations are prevented. This hypothesis is reinforced by the fact that, in both cases, decohesion decreased, meaning that the CA was effective, especially in the case of the green composite.

The results of the mechanical and AE analysis were validated using SEM images of the fractured face. These results showed that fibers were better embedded in the matrix upon the addition of CA, and the bio composite surface was more distorted at failure, which caused substantial AE activity during the damage process.

Despite its excellent mechanical behavior described in the literature coupled with other ecological benefits, the long-term applications of PE green material must be chosen carefully because bio and green composites with similar static properties exhibited different damage processes in tests, such as cycling and creep. AE equipment could be used to verify structured aging with regards to creep to predict when replacement is required and avoid catastrophic failures. For future studies, it is recommended to perform comparative fatigue testing on these materials. Frequential analysis of the damage processes using AE is also recommended.
